# Supplementary material for: A Novel Long Noncoding RNA in Osteocytes Regulates Bone Formation through the Wnt/β-Catenin Signaling Pathway
Source: Int J Mol Sci. 2023 Sep 4;24(17):13633. doi: 10.3390/ijms241713633 (PMC10488071; doi:10.3390/ijms241713633)
Supplement: Supplementary file 1 [file ijms-24-13633-s001.zip › ijms-2583444-supplementary.pdf]

Supplementary Materials

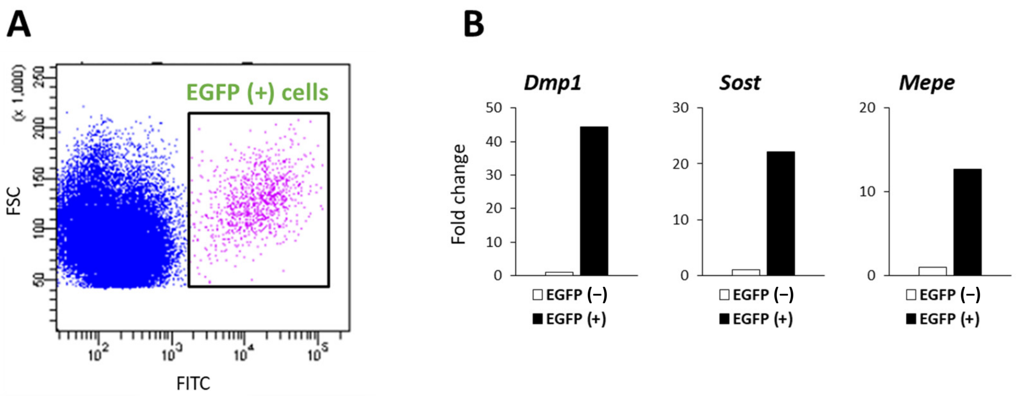

Supplementary Figure S1. Confirmation of the isolation of osteocytes. (**A**) Scatter plot of the cells derived from femurs of *Dmp1*-Cre;*CAG-CAT-EGFP tg* mice. These cells were analyzed by RNA-seq and the related heatmap is shown in Figure 1A. (**B**) Expression of osteocyte marker genes in RNA-seq samples.

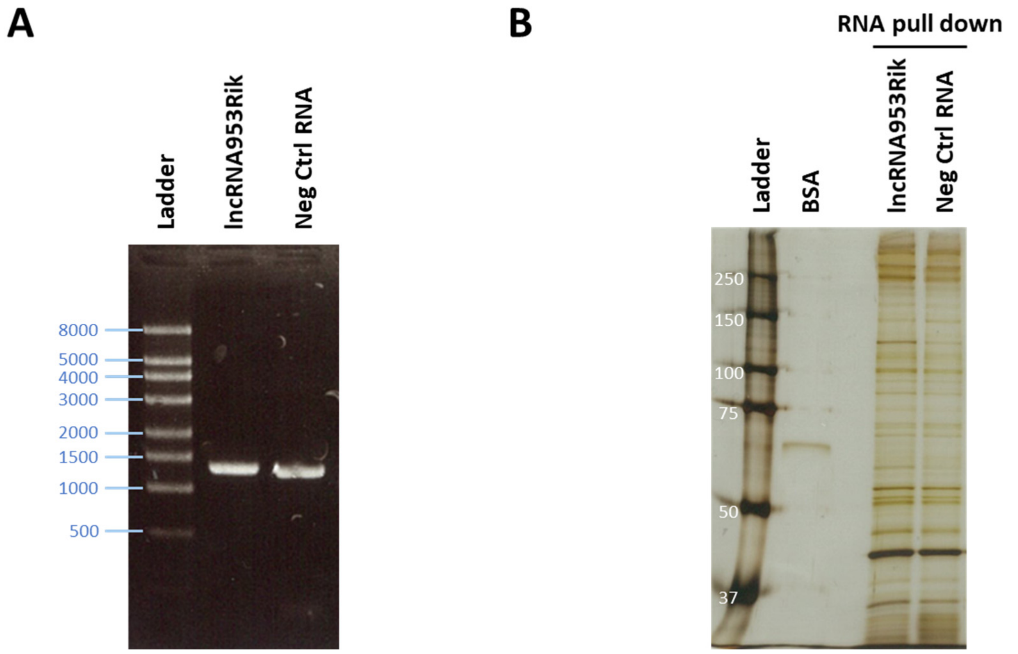

Supplementary Figure S2. RNA pull-down using in vitro-transcribed lncRNA953Rik. (**A**) Agarose gel electrophoresis of in vitro-transcribed lncRNA953Rik or a negative control RNA of the same length. These RNAs were used for the RNA pull-down assay. (**B**) In vitro-transcribed lncRNA953Rik or the negative control RNA was incubated with whole-cell lysates from IDG-SW3 cells. After pull-down of each RNA, the samples were separated by SDS–PAGE, and the gels were subjected to silver staining. BSA, bovine serum albumin (66 kDa).

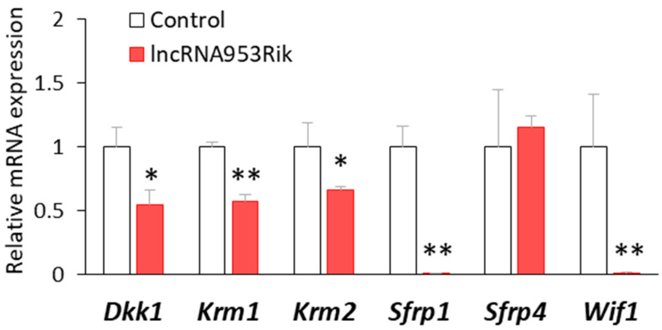

Supplementary Figure S3. Expression of Wnt/β-catenin signaling inhibitors. The expression of representative genes of Wnt/β-catenin signaling inhibitors was analyzed by qPCR in the absence of differentiation. The indicated mRNA expression levels were normalized to the level of *Gapdh*. Error bars, SDs; $n = 3$; t test; *, $p < 0.05$; **, $p < 0.01$ versus control.
